# Cardiac Events in Childhood Cancer Survivors Treated with Anthracyclines: The Value of Previous Myocardial Strain Measurement

**DOI:** 10.3390/life12030452

**Published:** 2022-03-19

**Authors:** Milanthy Pourier, Remy Merkx, Jacqueline Loonen, Alyssa van Cleef, Chris de Korte, Louise Bellersen, Livia Kapusta, Annelies Mavinkurve-Groothuis

**Affiliations:** 1 Department of Pediatrics Amalia Children’s Hospital, Radboud University Medical Center, 6500 HB Nijmegen, The Netherlands; 2 Department of Medical Imaging, Radboud University Medical Center, 6500 HB Nijmegen, The Netherlands; remy.merkx@radboudumc.nl (R.M.); chris.dekorte@radboudumc.nl (C.d.K.); 3 Department of Hematology, Radboud University Medical Center, 6500 HB Nijmegen, The Netherlands; jacqueline.loonen@radboudumc.nl (J.L.); alyssa.vancleef@radboudumc.nl (A.v.C.); 4 Department of Cardiology, Radboud University Medical Center, 6500 HB Nijmegen, The Netherlands; louise.bellersen@radboudumc.nl; 5 Pediatric Cardiology Unit, Tel Aviv Sourasky Medical Center, Sackler School of Medicine, Tel Aviv University, Tel Aviv 64239, Israel; livia.kapusta@radboudumc.nl; 6 Department of Pediatric Cardiology, Amalia Children’s Hospital, Radboud University Medical Center, 6500 HB Nijmegen, The Netherlands; 7 Princess Máxima Center for Pediatric Oncology, 3720 AC Utrecht, The Netherlands; a.m.c.mavinkurve-groothuis@prinsesmaximacentrum.nl

**Keywords:** cardiotoxicity, strain imaging, anthracyclines, cardiac event, heart failure, childhood cancer survivors, echocardiography, left ventricular dysfunction

## Abstract

In echocardiographic surveillance of anthracycline-treated childhood cancer survivors (CCS), left ventricular ejection fraction (LVEF) has insufficient prognostic value for future cardiac events, whereas longitudinal strain may be more sensitive. We describe the long-term incidence of cardiac events in CCS after previous measurement of LVEF and myocardial strain. Echocardiography, including four-chamber view longitudinal strain (4CH-LS), of 116 anthracycline-treated CCS was obtained between 2005–2009 (index echocardiography). Follow-up was obtained at the late-effects clinic. Primary outcome was occurrence of cardiac events, defined as either symptomatic heart failure, life-threatening arrhythmias, LVEF < 40% or cardiac death, in CCS with normal versus abnormal index 4CH-LS. LVEF from subsequent echocardiograms was obtained to evaluate its natural course as a secondary outcome. After index echocardiography (median 13.1 years since childhood cancer diagnosis), our study added a median follow-up of 11.3 years (median last clinical contact 23.6 years since diagnosis). Only three CCS developed a cardiac event (6.2, 6.4 and 6.7 years after index echocardiography), resulting in a ten-year cumulative incidence of 2.7% (95%CI 0.9–8.2). All three CCS had a clearly reduced index 4CH-LS and relevant cardiovascular risk factors, whereas their index LVEFs were around the lower limit of normal. Index LVEF correlated with index 4CH-LS but mean long-term natural course of LVEF was comparable for CCS with abnormal versus normal index 4CH-LS. Absolute 10-year cumulative incidence of cardiac events in anthracycline-treated CCS during long-term follow-up was low. Sensitive echocardiographic measurements, such as 4CH-LS may be useful to tailor surveillance frequency in a selected group of CCS without cardiovascular disease.

## 1. Introduction

The survival of children with cancer has improved over the last decades, reaching a 5-year survival of approximately 80% [1]. However, treatment-related cardiotoxicity results in a six-fold higher risk of cardiomyopathy or heart failure specific mortality in childhood cancer survivors (CCS), compared to the general population [2]. The estimated risk of symptomatic heart failure, 40 years after treatment with cardiotoxic chemotherapy, is 10.5% [3]. Addition of mediastinal radiotherapy to anthracyclines increases this risk to 27.8% [3]. Early detection of subclinical cardiotoxicity is crucial to facilitate timely intervention.

Echocardiographic surveillance is currently used to detect asymptomatic cardiac dysfunction [4,5]. However, a significant decrease in left ventricular ejection fraction (LVEF), the current standard to quantify systolic function, may be a late presentation of cardiotoxicity [6,7]. Moreover, LVEF on 2D echocardiography has been shown to have insufficient reproducibility, hampering serial assessments [8]. In the expert consensus for cardiac imaging in adult patients during and after cancer therapy, longitudinal myocardial strain is regarded as the single best parameter to predict anthracycline-induced cardiotoxicity [9]. A relative decrease from 10% to 15% of this parameter is often seen before a relevant reduction of LVEF is observed [4]. Evidence on the additional value of longitudinal strain in predicting chemotherapy-induced cardiotoxicity [10] and the prognostic implications of longitudinal strain in the general adult population with cardiovascular disease [11] is accumulating in adults.

The addition of echocardiographic measurements obtained during surveillance, to existing prediction models for heart failure in CCS that are only based on cardiotoxic exposures [6], may aid in the identification of CCS that need more or less frequent surveillance. Only recently it was shown that LVEF measurement, at first surveillance echocardiography in adulthood, reclassified a large group of CCS to having a low 10-year risk of relevant left ventricular dysfunction (defined as an LVEF < 40%). CCS with an initial mid-range LVEF (40–49%) had an almost eight-fold risk compared to those with a preserved LVEF (LVEF ≥ 50%) [12].

A recent systematic review showed that the use of longitudinal strain identifies a higher prevalence of asymptomatic left ventricular systolic dysfunction in anthracycline-treated CCS compared to the use of LVEF [13]. However, its prognostic implications in CCS remain unknown [5,14]. In the present study, we aimed to describe the incidence of cardiac events in CCS during long-term follow-up, a decade after an index echocardiography was made that included strain measurement.

## 2. Materials and Methods

### 2.1. Population

This retrospective cohort study adds follow-up to the cohort previously described by Mavinkurve-Groothuis et al. [15]. We included CCS who visited the late-effects clinic > 5 years after cancer diagnosis, from December 2005 until November 2009, and who received anthracyclines as part of their cancer therapy, with or without radiotherapy on the heart region. These CCS received cardiac follow-up at the late-effects clinic in accordance with current guidelines [5]. Exclusion criteria at baseline were clinical heart failure defined by the New York Heart Association classification (NYHA, class II–IV), a history of cardiovascular diseases or chronic renal insufficiency.

### 2.2. Cardiac Imaging

At study initiation, all participants underwent detailed transthoracic echocardiography (referred to as index echocardiography) at rest, according to the recommendations of the American Society of Echocardiography [16]. Images were obtained using a commercially available Vivid 7 echocardiographic scanner (GE, Vingmed Ultrasound, Horton, Norway). Systolic left ventricular function at the index echocardiography was conventionally indicated by measuring biplane LVEF (Simpson’s method) and fractional shortening. Details of our speckle-tracking based strain analysis methods have been described earlier by our group [17]. We measured longitudinal strain from an apical four-chamber view (4CH-LS) and circumferential strain from a parasternal mid-ventricular short axis view (mid-CS; papillary muscle level). A reduced index LVEF was defined below 52% and 54% for men and women, respectively, following the latest chamber quantification guidelines [15]. Myocardial strain measurements will be referenced as absolute values (i.e., −20% is ‘better’ and ‘higher’ than −18%). The thresholds for reduced longitudinal and circumferential strain at index echocardiography were defined by using sex-specific reference values of healthy adults [18]. For CCS in pediatric age at the index echocardiography, age-specific normative values were used [19].

### 2.3. Data Extraction and Outcomes

Using this study cohort, with the index echocardiography as the starting point for the current study, we collected medical record data up to the last clinical contact at our late-effects clinic or cardiology clinic before March 2021. To calculate cumulative anthracycline dose, we used a new dose-equivalence formula for cardiotoxicity, which includes daunorubicin, doxorubicin, epirubicin and idarubicin [20]. We separately reported on mitoxantrone exposure, since its cardiotoxic potential is of a higher magnitude than that of anthracyclines [20]. Data on prescribed total radiation dose on lung, mediastinal fields and total body irradiation were collected. Data were extracted on possible cardiac events during follow-up, as well as development of clinically diagnosed hypertension, diabetes mellitus, thyroid disease and dyslipidemia and smoking behavior either before or during follow-up. Moreover, LVEF measurement of the latest surveillance echocardiography was extracted (referred to as last echocardiography), and preferably also from an echocardiography between the index echocardiography and the latest echocardiography (referred to as middle echocardiography). The primary study endpoint was the occurrence of cardiac events, including heart failure diagnosed by a cardiologist, life-threatening arrhythmias, echocardiographic LVEF < 40% measured at any point in time during follow-up or cardiac death. Secondary outcome was the natural course of LVEF for CCS with normal versus abnormal index 4CH-LS. CCS were censored from this analysis at the time of occurrence of a cardiac event.

### 2.4. Statistical Analysis

Characteristics of the study population and echocardiographic data were summarized as median (range) or mean (standard deviation), as appropriate. Results on the occurrence of cardiac events remained descriptive; inferential statistics between CCS with normal versus abnormal 4CH-LS were not performed due to the low number of events. Missing values of 4CH-LS and LVEF were not imputed. Simple linear regression of index 4CH-LS and index LVEF was performed and significance of the correlation tested with ANOVA test. The mean natural course of LVEF was plotted against the time since index echocardiography using 5th order polynomials, constructed with a linear mixed-effects model. This model allowed inclusion of subjects with incomplete serial LVEF measurements and measurements with unequal time intervals and assigned a random intercept per subject. Differences in the natural LVEF course of CCS with normal versus abnormal index 4CH-LS were assessed with a likelihood ratio test, with a *p*-value < 0.05 considered statistically significant. Analyses were performed in SPSS version 25 (IBM Corp., Armonk, NY, USA).

## 3. Results

### 3.1. Population

**Figure 1:**
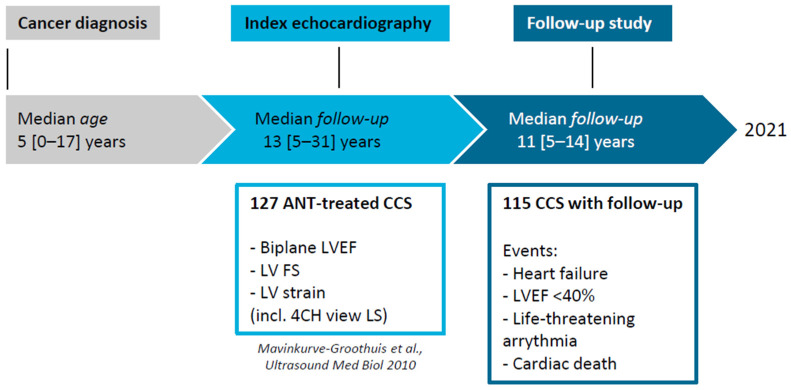
Study design and timeline. 4CH: apical 4-chamber view, EF: ejection fraction, FS: fractional shortening, LV: left ventricle, LS: longitudinal strain, ANT: anthracyclines, CCS: childhood cancer survivor.

The initial cohort included one hundred and twenty-seven CCS, of which eleven CCS were lost to follow-up for unknown reasons. Demographic characteristics, echocardiographic parameters at index echocardiography and at last clinical contact of the remaining 116 CCS are shown in Table 1. The majority of CCS were diagnosed with leukemia. All CCS received anthracyclines with a median cumulative anthracycline dose of 160 (range: 50–500) mg/m^2^. Eight CCS received mitoxantrone. Twelve CCS (10%) also received concurrent radiotherapy on mediastinal, lung or total body fields. At the index echocardiography, median time since childhood cancer diagnosis was 13.1 (range 4.9–30.8) years. Since then, the cohort was followed for a median of 11.3 years (range: 4.9–14.8), resulting in a median time since diagnosis of 23.6 years at last clinical contact. The study timeline is described in further detail in Figure 1.

### 3.2. Cardiac Events

**Figure 2:**
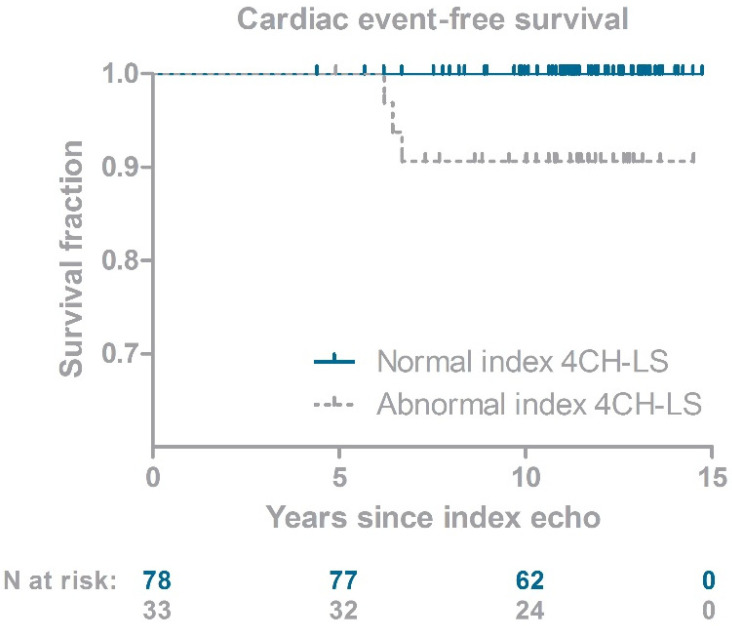
Development of cardiac events in childhood cancer survivors after index echocardiography. 4CH-LS: 4-chamber view longitudinal strain.

One hundred and eleven CCS had an available 4CH-LS at index echocardiography. We previously showed that 4CH-LS at index echocardiography was not correlated to age at diagnosis, follow-up duration or cumulative anthracycline dose [15]. In 33 CCS (30%) reduced 4CH-LS values were observed. An index LVEF was available for seventy-five CCS and was reduced in 19 (25%). During the follow-up, three CCS developed a cardiac event (10-year cumulative incidence since index echocardiography 2.7% (95%CI 0.9–8.2%). Two CCS presented with symptomatic heart failure, and one was detected with an asymptomatic LVEF < 40% at surveillance echocardiography. Detailed information on these three CCS is shown in Table 2. All three CCS received cumulative anthracycline doses > 200 mg/m^2^ and one of them received mediastinal radiotherapy. One CCS was a known smoker, one had hypothyroidism and one had a multinodular goiter and obesity (BMI 30.12 kg/m^2^). Two of three CCS had an elevated NT-pro-BNP at time of index echocardiography, according to normative values for adults [21] and children [22]. Figure 2 illustrates the occurrence of cardiac events over time.

All events occurred in CCS with a reduced index 4CH-LS, with an interval of six to seven years after index echocardiography. Consequently, none of the CCS with a normal 4CH-LS developed a cardiac event. The low number of events prevented statistical ana-lysis on the difference between CCS with normal versus abnormal index 4CH-LS. Table 2 furthermore shows that two of three CCS with a cardiac event had an index LVEF available. Both measured just around the lower limit of normal, whereas all three index GLS values were clearly abnormal. Fractional shortening on index echocardiography was >30% for all three cases.

### 3.3. Cardiac Imaging

**Figure 3:**
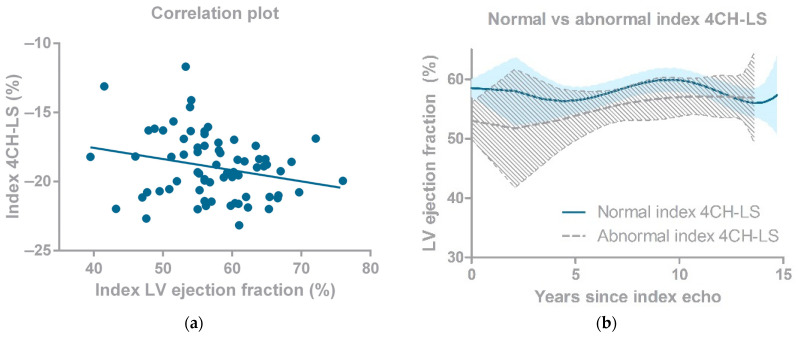
(**a**) Correlation plot 4CH-LS and LVEF; (**b**) Natural course of LVEF over time in CCS in normal versus abnormal 4 chamber view longitudinal strain. GLS: global longitudinal strain, LV: left ventricle, 4CH-LS: 4-chamber view longitudinal strain.

Index 4CH-LS and index LVEF were correlated (Figure 3a; β −0.081 per unit increase in LVEF, 95% CI: −0.157 to −0.005; *p* = 0.038). When comparing the natural course of LVEF values over time between CCS with normal and CCS with abnormal index 4CH-LS, LVEF values differed initially, but were comparable to each other after long term follow-up, as shown in Figure 3b.

## 4. Discussion

To our knowledge, this study was the first to assess the incidence of clinically important cardiac outcomes in anthracycline-treated CCS and relate these outcomes to previous myocardial strain measurements on a surveillance echocardiography during long-term follow-up. We analyzed a cohort of CCS that had an index echocardiography, with or without asymptomatic cardiac dysfunction, at a median of 13.1 years after cancer diagnosis and no cardiovascular disease at study initiation. During the subsequent median follow-up of 11.3 years, only three of the 116 CCS developed heart failure or a clinically relevant reduced LVEF, all > 5 years after index echocardiography. Therefore, the absolute 10-year cumulative incidence after index echocardiography was low. All three cases were identified by a clearly reduced 4CH-LS at the index echocardiography, combined with at least one relevant cardiovascular risk factor or comorbidity. The incidence of cardiac events in our cohort is not unexpected. In a large Dutch cohort, the 40-year cumulative incidence of heart failure after cancer diagnosis (grade 3 to 5 by definition of Common Terminology Criteria for Adverse Events) was found to be 10.5% in CCS exposed to cardiotoxic chemotherapy (anthracyclines or mitoxantrone). This cumulative incidence showed a quite stable slope over time, indicating that in any 10-year time interval until 40 years after diagnosis, an incidence around a fourth of this number can be expected [3]. Mulrooney et al. [23] recently reported the 15-year cumulative incidence of heart failure to be 0.69%, 0.74% and 0.54% for CCS treated in 1970–1979, 1980–1989 and 1990–1999, respectively. However, a considerable percentage of CCS in their cohort (49%) had not received anthracyclines, whereas anthracycline exposure constitutes a known and dose-dependent risk factor for developing cardiomyopathy in CCS [5,24]. This might explain the higher incidence in our study population.

In our cohort, we found 30% of CCS to have a decreased index 4CH-LS. This high prevalence corresponds well to the range of 27–40% (depending on the addition of chest radiotherapy to anthracycline exposure) found in a cohort including 1820 adult CCS in the United States [25]. Although we regard the subsequent absolute 10-year incidence of cardiac events to be low, we would not want to convey the message that cardiotoxicity is only a trivial concern in CCS exposed to cardiotoxic chemotherapy. We did not include a reference population, whereas it is known that CCS are at much higher risk of developing heart failure than their siblings [23]. However, combined with the recent findings by Lee-rink et al. that 75% of CCS have a very low 10-year risk of developing an LVEF < 40%, prediction of which is aided by the first LVEF measurement at long-term follow-up [12], our study suggests that a considerable proportion of CCS treated with anthracyclines may benefit from a lower surveillance frequency. In CCS with a normal index 4CH-LS, no cardiac events occurred. Notably, in the CCS who developed a cardiac event, the index LVEF was not clearly abnormal. This suggests that normal longitudinal strain in long-term CCS may predict a negligible incidence of cardiac events in the subsequent 10 years, which may even add to the predictive value of LVEF. This may lead to a more tailored advice for follow-up intervals periods in a selected group of CCS.

The suggestion that longitudinal strain may be a sensitive predictor of subsequent cardiac events is supported by the systematic review by Oikonomou et al. [10], that included 21 studies on the prognostic value of global longitudinal strain in adult cancer patients with a lower median follow-up duration (4.2 to 23.0 months). Studies in children reported a decrease of longitudinal strain parameters in children during cancer treatment [26] and further decline of strain parameters at late follow-up (>5 years) [17]. As we reported earlier, little research has been done on the prognostic value of strain in childhood cancer survivors. A recent review by Lenihan et al. from the global cardio-oncology summit defined the 10 top priorities to actualize for cardio-oncology with “defining robust predictors of cardiotoxicity” being one of them [27]. Assessing the possible added value of strain measurements in the prediction of cardiotoxicity in CCS fits this strategy.

Regarding the finding that the mean natural course of LVEF is similar for CCS with normal versus abnormal 4CH-LS, we have several hypotheses. Firstly, one should consider that biplane LVEF is known to have a high measurement error, possibly introducing high variability during serial assessments [9]. Second, the natural course of LVEF in CCS who developed a cardiac event could not be assessed after they were censored, introducing a small bias towards CCS without events. However, our main hypothesis remains that, as is also reflected in the low absolute 10-year incidence of cardiac events, the majority of CCS indeed has quite a stable natural course of LVEF. The number of CCS actually developing a significant LVEF decrease, often as a late sign of cardiotoxicity, may be too low to be reflected in the mean LVEF value. A study with a larger sample size may be able to detect more subtle differences in the natural LVEF course between CCS with normal and abnormal index 4CH-LS.

Limitations mostly seen in a retrospective study also apply to our study. Specifically, biplane LVEF could not be measured in all CCS. In particular, pediatric echocardiographies often lacked a two-chamber view, preventing calculation of biplane LVEF. Single-view longitudinal strain measurement in the four-chamber view was the standard protocol at the time of index echocardiography. Multiview global longitudinal strain has now been shown to have superior reproducibility [28]. The exact incidence date of the development of an asymptomatic LVEF < 40%, when detected at a surveillance echocardiography, remained unknown. Since our composite endpoint also included symptomatic events and since these CCS are regularly surveilled, we considered the date of echocardiography to be sufficiently detailed. Our study cohort only included 115 CCS, hampering statistical comparison of the few cardiac events in CCS with normal versus abnormal 4CH-LS and correction for confounding cardiotoxic exposures and traditional cardiovascular risk factors. The established larger national and international CCS cohorts may help to better statistically test the added value of myocardial strain measurement and combine these findings with the existing risk prediction models. However, most of these cohorts were established after our index echocardiography, which makes our current study unique for its follow-up duration.

## 5. Conclusions

During long-term follow-up of anthracycline-treated CCS without cardiovascular disease at baseline, the absolute 10-year cumulative incidence of cardiac events was low. None of the CCS with a normal index 4CH-LS developed a cardiac event and all cases with a cardiac event had an abnormal index 4CH-LS and at least one relevant cardiovascular risk factor. Therefore, addition of a sensitive tool such as 4CH-LS (e.g., measured five to ten years after diagnosis) to existing risk prediction models may aid to tailor the surveillance frequency in a selected group of CCS without cardiovascular disease.

## Figures and Tables

**Table 1:** Characteristics of 116 CCS in study cohort.

Demography and Cancer History	
Male sex, *n* (%)	68 (59)
Age at cancer diagnosis, years	4.5 (0.03–16.9)
Cancer diagnosis, n (%)	
Acute lymphoblastic leukemia	37 (32)
Acute myeloid leukemia	13 (11)
Lymphoma	30 (26)
Renal tumor	12 (10)
Neuroblastoma	9 (8)
Hepatic tumor	5 (4)
Bone tumor	7 (6)
Soft tissue sarcoma	3 (3)
Anthracycline exposure, n (%)	116 (100)
Cumulative anthracycline dose, mg/m^2^	160 (50–500)
<250 mg/m^2^	88 (76)
≥250 mg/m^2^	28 (24)
Mitoxantrone exposure, n (%)	8 (7)
Cumulative mitoxantrone dose, mg/m^2^	50 (20–122)
Mediastinal radiotherapy, n (%)	8 (7)
Mediastinal radiation dose, Gy	25 (12–35)
Lung radiotherapy, n (%)	2 (2)
Lung radiation dose, Gy	23 (15–30)
Total body irradiation, n (%)	2 (2)
Total body irradiation dose, Gy	12 (-)
**Index echocardiography**	
Age, years	18.5 [5.6–39.5]
Time since cancer diagnosis, years	13.1 [4.9–30.8]
Biplane LVES, %	57.3 (6.9)
Decreased LVEF, n (%)	19/75 (25)
4CH-LS, %	−18.5 (2.5)
Decreased 4CH-LS, n (%)	33/111 (30)
Mid-CS, %	−19.5 (2.9)
Decreased mid-CS, n (%)	11/88 (13)
Mitral valve E/A ratio	1.99 (0.64)
Fractional shortening, %	35.2 (3.9)
**Last clinical contact**	
Age, years	29.8 [14.5–50.9]
Body mass index, kg/m^2^	23.4 [17.0–44.3]
Systolic blood pressure, mmHg	121 (12)
Diastolic blood pressure, mmHg	74 (10)
Time since cancer diagnosis, years	23.6 [11.3–41.2]
Time since index echocardiography, years	11.3 [4.9–14.8]
Risk factors before/during follow-up, *n* (%)	
Hypertension	2 (2)
Overweight	29 (25)
Obesity	9 (8)
Diabetes mellitus	6 (5)
Dyslipidemia	5 (4)
Thyroid disease	10 (9)
Smoking	33 (28)

Continuous data are summarized as median (range), mean (SD). CCS: childhood cancer survivor; LVEF: left ventricular ejection fraction; 4CH-LS: 4-chamber view longitudinal strain; mid CS: mid ventricular circumferential strain.

**Table 2:** Characteristics of childhood cancer survivors who developed a cardiac event.

Case	Sex	Cardiac Event Type	Age at Event	Years since Diagnosis	Years since Index Echo	CAD (mg/m^2^)	MIT (mg/m^2^)	RT (Gy)	Risk Factors	LVEF	4CH-LS Index Echo	Mid-CS Index Echo
1	F	Symptomatic heart failure (palpitations at late effect clinic)	40	35.9	6.7	235	No	Mediastinal, 12	Multinodular goiterObesity	Index: -	−14.7	-
										Middle: 47%		
										Last: 50%		
2	M	Symptomatic heart failure (LVEF < 40% at surveillance echo, dyspnea on exertion in retrospect)	20	13.0	6.4	228	40	No	Smoking	Index: 52%	−15.7	−18.2
										Middle: 42%		
										Last: 48%		
3	M	LVEF < 40% at surveillance echo	22	16.3	6.2	360	40	No	Hypothyroidism	Index: 53%	−11.7	−14.5
										Middle: 55%		
										Last: 36%		

F: female; M: male; CAD: cumulative anthracycline dose; MIT: cumulative mitoxantrone dose; RT: radiotherapy; Gy: Gray; LVEF: left ventricular ejection fraction; 4CH-LS: 4-chamber view longitudinal strain; Mid-CS: mid ventricular circumferential strain.

## Data Availability

The datasets used and/or analyzed during the current study are available from the corresponding author on reasonable request.
